# Effect of COVID-19 pandemic on eating habits and lifestyle of college students in Tabriz, Iran: a cross-sectional study

**DOI:** 10.3389/fpubh.2023.1185681

**Published:** 2023-08-03

**Authors:** Maryam Rafraf, Roghayeh Molani-Gol, Mina Sahebjam

**Affiliations:** ^1^Nutrition Research Center, Department of Community Nutrition, Faculty of Nutrition and Food Science, Tabriz University of Medical Sciences, Tabriz, Iran; ^2^Student Research Committee, Tabriz University of Medical Sciences, Tabriz, Iran

**Keywords:** coronavirus, COVID-19, eating habits, lifestyle, college students

## Abstract

**Background:**

The pandemic of coronavirus disease (COVID-19) has influenced lifestyle behaviors and the health of populations worldwide. The purpose of this study was to determine the effects of the COVID-19 pandemic on the eating habits and lifestyle behaviors of Tabriz University of Medical Sciences students in Tabriz, Iran.

**Methods:**

This cross-sectional study was conducted on 220 college students selected using a convenience sampling method in May–June 2022. Data were collected by the questionnaire, which included information on eating habits, physical activity, smoking, watching television, social media use, sleep, anxiety and stress, and smoking before and during the pandemic. The Chi-square test was used to analyze the association of COVID-19 with lifestyle behaviors.

**Results:**

The median age of participants was 22.00 (IQR: 3.00) years old. The median BMI was 21.69 (IQR: 3.82) kg/m^2^, and 74.5% of participants had a BMI of 18.5 to 25 kg/m^2^. Around 34.5% of participants reported a weight gain during the pandemic. During the pandemic, students’ eating habits improved by maintaining a regular meal pattern, eating a balanced diet, consuming 2–3 servings of milk or its products, consuming one or more servings of pulses, eggs, or meat per day, decreasing consumption of fast food, fried, and junk foods, adding less sugar to meals and beverages, and consuming fewer sugar-sweetened beverages and foods with high sugar (all *p* = 0.000). They also reported less physical activity and more sitting and screen time. Sleep time and poorer quality of sleep increased during the pandemic (*p* = 0.000). Feeling stress or anxiety in a day increased, and 2.2% of our participants decided to smoke. The biggest reasons for eating habits changes were less eating out, fear of coronavirus spreading through food, preferring home-cooked food, and improved knowledge about nutrition.

**Conclusion:**

The results indicated that the eating habits of university students improved; however, participants stated increased weight gain, screen, sitting, and sleep time, declined physical activity, worse sleep quality, and feeling stress or anxiety during the COVID-19 pandemic. The findings can help to develop nutritional and behavioral recommendations for maintaining adults’ health during and after the pandemic.

## Introduction

Coronavirus disease 2019 (COVID-19) is an acute respiratory syndrome that its outbreak has been the most serious health threat globally in recent years (1). The World Health Organization (WHO) on March 11, 2020, stated that COVID-19 is a pandemic and declared it a “health emergency” due to the widespread massively in almost every country in the world (2). It is demonstrated that eating habits and lifestyle of individuals could be influenced by various diseases such as obesity. Worries about the one’s health contribute to greater healthcare and healthier lifestyle, on the other hand, emotional and physical consequence of disease might lead to the poor lifestyle patterns (3, 4). The COVID-19 pandemic also leads to unprecedented health, environmental, and economic crises (5). It has negatively impacted mental health, education, social life, and physical activity and overwhelmed the healthcare systems (6–8).

During the COVID pandemic, countries developed several strategies such as lockdowns for the avoidance of mass gatherings that forced the total population into home confinement and the academic community promptly switched to teleworking. Numerous changes in lifestyle habits such as eating habits and physical activity have been induced by the lockdowns (9). These changes may contribute to unhealthy behaviors, including poor diet, sedentariness or less physical activity, disturbed sleep patterns, and distress and anxiety that can potentially lead to obesity and its related cardiometabolic risks (10). Evidence of past outbreaks indicated that as a pandemic evolves, it substantially impacts lifestyle-related behaviors and poses a challenge to maintaining health and nutritional status (11). Thus, this pandemic has extensive medical, behavioral, and social implications. Understanding the extent of lifestyle-related changes and their contributing COVID-19-specific reasons is important to prevent these changes and maintain individual and community health (12).

Recent studies have assessed how the eating behaviors and lifestyles of individuals have altered during the lockdowns, some of them indicating general trends toward weaker lifestyles and the adaption of unhealthy eating behaviors (13–16). Despite this, some others reported higher general population adherence to the Mediterranean diet as a healthy eating pattern during the lockdown (17–19). However, some of these studies had methodological limitations such as non-validated data collection tools and less representative samples. Moreover, the influences of COVID-19 infection on lifestyle-related behaviors may vary from country to country because of different cultural, social, and economic constructs (20). Some of recent studies reported that women more affected by the restrictions because of COVID-19 pandemic (21–23). In Iran, there is a lack of research examining the COVID-19 pandemic’s impacts on lifestyle habits. It is required to explore the dietary habits, and lifestyle behaviors among university students in the Iranian context. Addressing the information gaps will provide the government, public health stakeholders, and higher education sector baseline data needed to properly plan and implement lifestyle related policies for young adults during quarantine and future outbreaks.

To the best of our knowledge, no study has assessed the eating habits and lifestyle changes of Iranian college students during the COVID-19 lockdown. This study aimed to assess the COVID-19 pandemic’s impacts on the eating habits and lifestyle behaviors of college students by interview method and using a validated questionnaire in Tabriz, Iran.

## Materials and methods

### Study design and participants

The present cross-sectional study was carried out among female students at Tabriz University of Medical Sciences from May to June 2022. The inclusion criteria were females in the age of more than 18 years old, being student in Tabriz University of Medical Sciences, and willingness to participate in the study. All female medical sciences students from Tabriz University of Medical Sciences who agreed to participate in this study met the inclusion criteria. There was no restriction on the socioeconomic level, nationality, or occupation of the participants. The sample size was calculated based on the prevalence of the increasing in the consumption of chocolate and salty snacks (28%) reported by Celorio-Sardà et al. (24) and considering a 95% confidence level, a power of 90% in two tailed tests using G-Power software was calculated to be 211 subjects. Finally, 220 college students were chosen using the convenience sampling method. All participants, before completing the questionnaire, were informed of the study procedure and signed a written informed consent. This study protocol was approved by the Ethics Committee of Tabriz University of Medical Sciences (ethical code: IR.TBZMED.REC.1401.597).

### Data collection

We used the questionnaire developed and validated by Chopra et al. among adults (12). They developed a questionnaire to assess the effects of COVID-19 on lifestyle behaviors, such as eating habits. We only removed one item from their questionnaire about drinking alcohol because our participants were females, and in our country, females do not drink or smoke. The items of the questionnaire are presented in Tables 1–5. Four sections are included in the questionnaire. Section A consisted of socio-demographic and anthropometric parameters, and Sections B and C had twenty-three items for evaluating the changes in eating, physical activity, and sleep behaviors before and during COVID-19, respectively. In addition, Section D involved six items for investigating COVID-19-specific reasons for the change of lifestyle. The questionnaire was in paper form. Body mass index value (BMI) was obtained by self-reported weight and height, and participants with BMI = 18.5 to 25 kg/m^2^, BMI < 18.5 kg/m^2^, BMI = 25 to 30 kg/m^2^, and BMI > 30 kg/m^2^ were categorized as normal weight, underweight, overweight, and obese, respectively.

**Table 1:** Baseline characteristics of participants (*n* = 220).

Characteristics	Median	IQR
Age (year)	22	3
Weight (kg)	59	12.15
Height (cm)	164	9
BMI (kg/m^2^)	21.69	3.82
	**n**	**%**
*BMI (kg/m^2^)*
BMI < 18.5	22	10
BMI = 18.5 to 25	164	74.5
BMI = 25 to 30	28	12.7
BMI > 30	6	2.7
*Marital status*
Married	16	7.3
Single	204	92.7
*Field of study*
Medicine	48	21.8
Pharmacology	26	11.8
Nutrition	21	9.5
Laboratory	8	3.6
Nursing	23	10.5
Midwifery	7	3.2
Health	25	11.4
Anesthesiology	3	1.4
Information technology	8	3.6
Occupational therapy	4	1.8
Surgical technologist	3	1.4
Management of healthcare services	6	2.7
Informatics	4	1.8
Speech therapy	5	2.3
Nanomedicine	6	2.7
Physiotherapy	4	1.8
Radiology	2	0.9
Food sciences	2	0.9
Food Industry Engineering	3	1.4
Audiology	2	0.9
Dentistry	2	0.9
Bacteriology	2	0.9
Anatomy	4	1.8
Physiology	4	1.8
Grade
BSc	115	52.3
MSc	18	8.2
PhD	82	37.3
Prof	5	2.3
Characteristics	Median	IQR	*Weight gain during the pandemic*
No, my weight is stable	88	40
No, I think I lost weight	46	20.9
Yes, I think I gained some weight	76	34.5
I do not know	10	4.5

BMI, body mass index; IQR, interquartile range.

**Table 2:** Eating habits changes in before and during COVID-19.

Items	Pre-COVID-19 *n* (%)	During COVID-19 *n* (%)	Chi-square, df	*p*-value*	*p*-value**
How often did you maintain a regular meal pattern?			191.609, 16	**0.000**	0.85
a. Not routinely	38 (17.3)	24 (10.9)			
b. One to two times a week	29 (13.2)	44 (20.0)			
c. Three to four times a week	50 (22.7)	49 (22.3)			
d. Five to six times a week	19 (8.6)	39 (17.7)			
e. Almost daily	84 (38.2)	64 (29.1)			
How often did you consume fast food like pizza, burger, pasta or noodle as snacks or meals?			57.301, 12	**0.000**	0.795
a. Not routinely	82 (37.3)	100 (45.5)			
b. One to two times a week	119 (54.1)	82 (37.3)			
c. Three to four times a week	18 (8.2)	32 (14.5)			
d. Five to six times a week	1 (0.5)	5 (2.3)			
e. Almost daily	0 (0.0)	1 (0.5)			
How often did you consume fried food (fried bread/poori, fried snack such as fries)?	22 (10.0)	42 (19.1)	125.678, 12	**0.000**	0.113
a. Not routinely	119 (54.1)	103 (46.8)			
b. One to two times a week	66 (30.0)	58 (26.4)			
c. Three to four times a week	13 (5.9)	15 (6.8)			
d. Five to six times a week	0 (0.0)	2 (0.9)			
e. Almost daily					
How often did you consume junk foods (like popcorn, chips etc.) as snacks?			289.273, 16	**0.000**	**0.025**
a. Not routinely	58 (26.4)	98 (44.5)			
b. One to two times a week	117 (53.2)	72 (32.7)			
c. Three to four times a week	36 (16.4)	35 (15.9)			
d. Five to six times a week	6 (2.7)	12 (5.5)			
e. Almost daily	3 (1.4)	3 (1.4)			
What was the frequency of your fruits and vegetables intake?			225.964, 16	**0.000**	0.385
a. Not routinely	3 (1.4)	8 (3.6)			
b. One to two times a week	50 (22.7)	56 (25.5)			
c. Three to four times a week	80 (36.4)	66 (30.0)			
d. Five to six times a week	36 (16.4)	40 (18.2)			
e. Almost daily	51 (23.2)	50 (22.7)			
How often did you have a balanced diet by including healthy ingredients (whole wheat, pulses, legumes, eggs, nuts, fruits and vegetables) in your meals?			151.763, 16	**0.000**	0.058
a. Not routinely	15 (6.8)	10 (4.5)			
b. One to two times a week	60 (27.3)	59 (26.8)			
c. Three to four times a week	75 (34.1)	70 (31.8)			
d. Five to six times a week	35 (15.9)	39 (17.7)			
e. Almost daily	35 (15.9)	42 (19.1)			
How often did you have 2–3 servings of milk or its products (curd, buttermilk, cheese, paneer etc.) in a day?			233.074, 16	**0.000**	0.765
a. Not routinely	24 (10.9)	19 (8.6)			
b. One to two times a week	58 (26.4)	70 (31.8)			
c. Three to four times a week	74 (33.6)	65 (29.5)			
d. Five to six times a week	31 (14.1)	31 (14.1)			
e. Almost daily	33 (15.0)	35 (15.9)			
How often did you have one or more servings of pulses, egg or meat in a day?			224.817, 16	**0.000**	0.15
a. Not routinely	4 (1.8)	4 (1.8)			
b. One to two times a week	31 (14.1)	35 (15.9)			
c. Three to four times a week	106 (48.2)	79 (35.9)			
d. Five to six times a week	34 (15.5)	60 (27.3)			
e. Almost daily	45 (20.5)	42 (19.1)			
How many teaspoons of sugar/honey/jaggery did you consume in a day?			257.785, 16	**0.000**	0.786
a. Zero teaspoons per day, I do not add sugar in my meals/ beverages	64 (29.1)	72 (32.7)			
b. One to two teaspoons per day	87 (39.5)	74 (33.6)			
c. Three to four teaspoons per day	49 (22.3)	49 (22.3)			
d. Five to six times teaspoons per day	14 (6.4)	19 (8.6)			
e. More than 6 teaspoons per day	6 (2.7)	6 (2.7)			
	0 (0.0)	0 (0.0)			
How often did you consume sugar-sweetened beverages (juice, soft drinks, flavored soda etc.)?			105.825, 16	**0.000**	0.387
a. Not routinely	69 (31.4)	77 (35.0)			
b. One to two times a week	101 (45.9)	93 (42.3)			
c. Three to four times a week	32 (14.5)	33 (15.0)			
d. Five to six times a week	14 (6.4)	15 (6.8)			
e. Almost daily	4 (1.8)	2 (0.9)			
How often did you consume foods with high sugar (such as sweet porridges, pastries, sweets and chocolate etc.)?			193.373, 16	**0.000**	**0.049**
a. Not routinely	37 (16.8)	41 (18.6)			
b. One to two times a week	97 (44.1)	103 (46.8)			
c. Three to four times a week	52 (23.6)	50 (22.7)			
d. Five to six times a week	22 (10.0)	18 (8.2)			
e. Almost daily	12 (5.5)	8 (3.6)			
How often did you eat junk food/fast food due to boredom/distress/disappointment?			114.037, 16	**0.000**	0.063
a. Not routinely	137 (62.3)	135 (61.4)			
b. One to two times a week	65 (29.5)	53 (24.1)			
c. Three to four times a week	14 (6.4)	24 (10.9)			
d. Five to six times a week	2 (0.9)	5 (2.3)			
e. Almost daily	2 (0.9)	3 (1.4)			

Data are presented as frequency [percent (%)].

* Chi-square test.

** Wilcoxon Signed-Rank test, *p* < 0.05 is significant (the bold values).

**Table 3:** Physical activity changes in before and during COVID-19.

Items	Pre-COVID-19 *n* (%)	During COVID-19 *n* (%)	Chi-square, df	*p*-value*	*p*-value**
How often did you participate in 30 min of moderate intensity aerobic exercises/sports?			116.725, 16	**0.000**	0.216
a. Not routinely	37 (16.8)	80 (36.4)			
b. One to two times a week	97 (44.1)	67 (30.5)			
c. Three to four times a week	52 (23.6)	45 (20.5)			
d. Five to six times a week	22 (10.0)	16 (7.3)			
e. Almost daily	12 (5.5)	12 (5.5)			
How often did you participate in household chores (cooking, laundry, cleaning)?			188.215, 16	**0.000**	**0.046**
a. Not routinely	137 (62.3)	31 (14.1)			
b. One to two times a week	65 (29.5)	75 (34.1)			
c. Three to four times a week	14 (6.4)	56 (25.5)			
d. Five to six times a week	2 (0.9)	26 (11.8)			
e. Almost daily	2 (0.9)	32 (14.5)			
How often did you participate in leisure related activities (grocery shopping, walking in park, gardening)?			122.501, 16	**0.000**	**0.002**
a. Not routinely	28 (12.7)	49 (22.3)			
b. One to two times a week	97 (44.1)	101 (45.9)			
c. Three to four times a week	59 (26.8)	39 (17.7)			
d. Five to six times a week	23 (10.5)	20 (9.1)			
e. Almost daily	13 (5.9)	11 (5.0)			
How much was your daily sitting time at work?			61.961, 16	**0.000**	0.408
a. Less than 2 h	30 (13.6)	50 (22.7)			
b. 2–4 h	43 (19.5)	47 (21.4)			
c. 4–6 h	76 (34.5)	42 (19.1)			
d. 6–8 h	47 (21.4)	39 (17.7)			
e. More than 8 h	24 (10.9)	42 (19.1)			
How many breaks from sitting (such as standing up, or stretching or taking a short walk) during your office hours did you typically take at work?			87.560, 16	**0.000**	0.555
a. 0	41 (18.6)	48 (21.8)			
b. 1–2	92 (41.8)	95 (43.2)			
c. 3–4	55 (25.0)	43 (19.5)			
d. 5–6	21 (9.5)	15 (6.8)			
e. More than 6	11 (5.0)	19 (8.6)			
How much screen time did you spend daily for watching television, using social media, mobile phones and playing video games?			71.715, 16	**0.000**	**0.000**
a. 0–1 h	22 (10.0)	15 (6.8)			
b. 1–2 h	55 (25.0)	35 (15.9)			
c. 2–4 h	104 (47.3)	65 (29.5)			
d. >5 h	39 (17.8)	105 (47.8)			

Data are presented as frequency [percent (%)].

* Chi-square test.

** Wilcoxon Signed-Rank test, *p* < 0.05 is significant (the bold values).

**Table 4:** Sleep changes and anxiety in before and during COVID-19.

Items	Pre-COVID-19 *n* (%)	During COVID-19 *n* (%)	Chi-square, df	*p*-value*	*p*-value**
How many hours did you sleep daily?			76.569, 4	**0.000**	**0.000**
a. <6 h	20 (9.1)	14 (6.4)			
b. 6–8 h	156 (70.9)	105 (47.7)			
c. >8 h	44 (20.0)	101 (45.9)			
How would you rate your quality of sleep?			165.795, 16	**0.000**	**0.000**
a. Excellent	35 (15.9)	30 (13.6)			
b. Very good	67 (30.5)	42 (19.1)			
c. Good	109 (49.5)	101 (45.9)			
d. Bad	7 (3.2)	39 (17.7)			
e. Very bad	2 (0.9)	8 (3.6)			
How much stress or anxiety did you feel in a day?			99.360, 16	**0.000**	**0.000**
a. Not at all	19 (8.6)	12 (5.5)			
b. A little	133 (60.5)	70 (31.8)			
c. Much	49 (22.3)	94 (42.7)			
d. Very much	14 (6.4)	32 (14.5)			
e. Extremely	5 (2.3)	12 (5.5)			
Did you smoke?			81.239, 2	**0.000**	**0.034**
a. No	218 (99.1)	214 (97.3)			
b. Yes, 1–3 cigarettes per day	2 (0.9)	4 (1.8)			
c. Yes, 4–6 cigarettes per day	0 (0.0)	0 (0.0)			
d. Yes, 7–9 cigarettes per day	0 (0.0)	0 (0.0)			
e. Yes, >10 cigarettes per day	0 (0.0)	0 (0.0)			
Did your family and friends support you to maintain a healthy lifestyle?			232.322, 16	**0.000**	0.243
a. Always (more than 90% times)	76 (34.5)	85 (38.6)			
b. Most of the time (approx. 75% times)	80 (36.4)	79 (35.9)			
c. Sometimes (approx. 50% times)	36 (16.4)	26 (11.8)			
d. Occasionally (approx. 25% times)	19 (8.6)	24 (10.9)			
e. Rarely (less than equal to10% times)	9 (4.1)	6 (2.7)			

Data are presented as frequency [percent (%)].

* Chi-square test.

** Wilcoxon Signed-Rank test, *p* < 0.05 is significant (the bold values).

**Table 5:** Reasons for lifestyle changes during COVID-19 pandemic.

Items	*n*	%
*What are the reasons for changes in dietary pattern in comparison to pre-COVID-19 times?*
a. Improved knowledge about nutrition	30	13.6
b. Lack of access to fresh fruits and vegetables	7	3.2
c. Higher cost of ingredients	18	8.2
d. More available cooking time	18	8.2
e. Better family support	16	7.3
f. Less eating out	63	28.6
g. Lack of family support	0	0
h. Stress and anxiety	19	8.6
i. Relaxed mind	6	2.7
j. No change	39	17.7
k. Any other, please specify_	4	1.8
*What are the reasons for changes in junk food/fast food consumption pattern in comparison to pre-COVID-19 times?*
a. Fear of coronavirus spread through food	57	25.9
b. Non-availability of cook	7	3.2
c. Less eating out/socializing	45	20.5
d. Availability of cooking time	13	5.9
e. Preferring home cooked food	42	19.1
f. Focus on eating healthy to build immunity	26	11.8
g. Managing food craving using different techniques such as listening to songs, taking awake	1	0.5
h. Lack of family support	1	0.5
i. Stress and/or anxiety	12	5.5
j. Any other, please specify	16	7.3
*In order to increase your physical activity, which activities have you included?*
a. At-home aerobics	15	6.8
b. Yoga	2	0.9
c. At-home workout videos	46	20.9
d. Gyming (treadmill, cycle and weights)	26	11.8
e. Walks	60	27.3
f. At-home dancing and stretching	38	17.3
g. Not doing any activities	29	13.2
h. Any other, Please specify	4	1.8
*What are the reasons for your change in physical activity regime during COVID-19?*
a. Lack of motivation	59	26.8
b. Lack of knowledge of exercises	4	1.8
c. Lack of access to sport facilities and gym	57	25.9
d. Social restrictions to parks and public places	37	16.8
e. Lack of social support	2	0.9
f. Lack of time	28	12.7
g. Any other, Please specify	33	15
*What are the reasons for a change in sleeping pattern during COVID-19?*
a. Daytime sleeping	79	35.9
b. Stress and anxiety	60	27.3
c. Long working hours	18	8.2
d. Environmental factors such as noise and lighting	8	3.6
e. Shortness of breath during sleep	3	1.4
f. Flexibility in days’ time	13	5.9
g. Any other, please specify	39	17.8
*What are the reasons for a change in stress and anxiety levels during COVID-19?*
a. Fear of COVID infection	45	20.5
b. Worrying about family and friends	99	45
c. Stigma or discrimination from other people (e.g., people treating you differently because of your identity, having symptoms, or other factors related to COVID-19)	4	1.8
d. Frustration/boredom/loneliness	37	16.8
e. Financial loss	3	1.4
f. Confusion about what COVID-19 is, how to prevent it, or why social	11	5
distancing/isolation/quarantines are needed	3	1.4
g. Lack of support from family and friends	18	8.2
h. Any other, please specify	0	0

Data are presented as frequency ± percent (%). *p < 0.05, Chi-square test.

### Statistical analysis

Data analysis was done using IBM SPSS version 26 software. The normality of continuous data was checked by the distribution of the data and the Kolmogorov–Smirnov test. Based on these methods, the distribution of our data was non-normal; hence, the median and interquartile range (IQR) was reported for continuous data. Categorical data were reported as frequency and percentage. The chi-square test the Wilcoxon Signed-Rank test were used to compare two scores before and during the COVID-19 pandemic for each item. A value of *p* < 0.05 was set as the significance level.

## Results

A total of 220 female students from a medical university participated in the current study. The general characteristics of the participants are presented in Table 1. The median age of the students was 22.00 (IQR: 3.00) years old. The majority were unmarried (92.7%), and 21.8% of them were medical students. Most respondents were BSc students (52%), and 37% were PhD students. In terms of BMI, the median was 21.69 (IQR: 3.82) kg/m^2^, and 10.0, 74.5, 12.7, and 2.7% of participants had BMI < 18.5, BMI = 18.5 to 25, BMI = 25 to 30, and BMI > 30, respectively. In addition, 40% of respondents reported that their weight was stable during the COVID-19 pandemic, while 34.5% of participants stated that they had gained weight during this time.

### Effects of the COVID-19 pandemic on the eating habits of participants

As shown in Table 2, the students’ eating habits significantly changed during the COVID-19 pandemic compared to before the pandemic. Having a regular meal pattern (Chi-square = 191.609, df = 16, *p* = 0.000), a balanced diet with healthy ingredients (Chi-square = 151.763, df = 16, *p* = 0.000), 2–3 servings of dairy products (Chi-square = 233.074, df = 16, *p* = 0.000), and one or more servings of pulses, meat, or egg daily five to six times a week (Chi-square = 224.817, df = 16, *p* = 0.000) all increased during the COVID-19 pandemic. Consuming fast food as snacks or meals (like pasta, pizza, noodle, or burgers; Chi-square = 57.301, df = 12, *p* = 0.000), consuming fried food (Chi-square = 125.678, df = 12, *p* = 0.000), consuming junk foods (like chips, popcorn, etc.) as snacks (Chi-square = 289.273, df = 16, *p* = 0.000), not adding sugar in meals/beverages (Chi-square = 257.785, df = 16, *p* = 0.000), consuming sugar-sweetened beverages (Chi-square = 105.825, df = 16, *p* = 0.000), and consuming foods with high sugar (Chi-square = 193.373, df = 16, *p* = 0.000) and eating junk food/fast food one to two times a week (Chi-square = 114.037, df = 16, *p* = 0.000) decreased during the pandemic. Based on the Wilcoxon Signed-Rank test results, not routinely consumption of junk foods (like popcorn, chips etc.) as snacks was increased during the COVID-19 (44.5%) compared to the before of the pandemic (26.4%; *p* = 0.025). Also, not routinely consumption of foods with high sugar (such as sweet porridges, pastries, sweets and chocolate etc.) also increased during the COVID-19 pandemic (18.6% vs. 16.8%, *p* = 0.049) (Table 2).

### Effects of the COVID-19 pandemic on the lifestyle of participants

Table 3 shows that participants’ physical activity changed significantly before and during COVID-19. In this way, participation in moderate-intensity aerobic exercises for 30 min (Chi-square = 116.725, df = 16, *p* = 0.000) and leisure-related activities (grocery shopping, walking in the park, and gardening; Chi-square = 122.501, df = 16, *p* = 0.000) decreased. While performing household chores (cleaning, cooking, and laundry; Chi-square = 188.215, df = 16, *p* = 0.000), sitting time at work of less than 2 h per day (Chi-square = 61.961, df = 16, *p* = 0.000), not breaking from sitting (for example, stretching, taking a short walk, or standing up) during office hours (Chi-square = 87.560, df = 16, *p* = 0.000), and spending more than 5 h per day on social media, mobile phones, watching television, and playing video games (Chi-square = 71.715, df = 16, *p* = 0.000) were increased. The Wilcoxon Signed-Rank test results showed that not participating in household chores (cooking, laundry, cleaning) was decreased (14.1% vs. 62.3%, *p* = 0.046), while, not participating in leisure related activities (grocery shopping, walking in park, gardening) was increased (22.3% vs. 12.7%, *p* = 0.002) during the COVID-19 compare to the before. In addition, >5 h screen time spend daily for watching television, using social media, mobile phones and playing video games was significantly increased during the pandemic (47.8% vs. 17.8%, *p* = 0.000; Table 3).

Sleep changes and anxiety before and during COVID-19 are indicated in Table 4. Their sleep time and quality significantly changed during the pandemic (all *p* = 0.000). Having >8 h of sleep daily was enhanced during the COVID-19 pandemic (Chi-square = 76.569, df = 4, *p* = 0.000). The students that have a very good quality of sleep decreased during the pandemic, whereas those who have a very bad quality of sleep increased during the pandemic compared to before (Chi-square = 165.795, df = 16, *p* = 0.000). Feeling very much stress or anxiety in a day was increased (Chi-square = 99.360, df = 16, *p* = 0.000), and no smoking was decreased during the COVID-19 pandemic, and 2.2% of our participants decided to smoke (Chi-square = 81.239, df = 16, *p* = 0.000). Family and friends’ support for maintaining a healthy lifestyle has increased during the COVID-19 pandemic compared to before the pandemic (Chi-square = 232.322, df = 16, *p* = 0.000). According to the Wilcoxon Signed-Rank test, except for the support of family and friends for maintaining a healthy lifestyle, sleep quality and stress and anxiety adversely influenced by the pandemic (all *p* < 0.05; Table 4).

### The lifestyle changes’ reasons during the COVID-19 pandemic

Reasons for lifestyle changes during the pandemic of COVID-19 are shown in Table 5. The biggest reasons for dietary pattern changes compared to pre-COVID-19 times were less eating out and improved knowledge about nutrition, and the most reasons for junk food and fast-food consumption changes in comparison to pre-pandemic times were fear of coronavirus spread via food, less eating out and socializing, and preferring home-cooked food. To increase physical activity, students included walks and at-home workout video activities, and the reasons for their physical activity levels changing during COVID-19 were a lack of motivation and access to sports facilities and a gym. The reasons for changes in sleeping patterns during COVID-19 were daytime sleeping and stress and anxiety. The reasons for changes in anxiety and stress levels during COVID-19 were worrying about family and friends and fear of COVID-19 infection.

## Discussion

The COVID-19 pandemic brought about significant changes in people’s lifestyles, diets, and psycho-emotional behaviors (25). Diet, exercise, physical activities, and even harmful habits (e.g., alcohol, caffeine-rich drinks, sugar-rich drinks, and smoking) are all components of an individual’s lifestyle (26, 27). The current study sought to assess the effects of the COVID-19 pandemic on college students’ eating habits, physical activities, sleep quality, anxiety, and stress. Majority of the included participants had a normal BMI; however, 34.5% of them reported that they gained weight during the COVID-19 pandemic. Similar to our findings, the study by Cheikh Ismail et al. indicated that 32.8% of the adults gained weight during the lockdown (28). Chen et al. also found that the college students reported a ≈ 3.4 kg weight gain after the first month of lockdown due to the pandemic (29). Rafique et al. determined that 48% of the young adults gained weight during the pandemic (30). Similarly, two meta-analyses assessing nearly 100 studies revealed that lockdowns during the COVID-19 pandemic increased the body weight among a high number of young adults (11.1–72.4%) (31, 32). The primary cause of enhanced BMI among young adults was the shift to online education which increased screen time, sitting hours, sleeping hours, food consumption, snacking, and cooking (29–32). Robinson et al. also demonstrated that higher BMI was associated with lower physical activity levels, poor diet quality, and frequent overeating (33).

According to the results of this study, the eating habits of students were characterized by an increase in having a regular meal pattern and balanced diet, milk or its products, pulses, and egg or meat in the diet, as well as by a decrease in consuming fast food, fried food, junk foods (e.g., potato chips, popcorn, etc.), sugar-sweetened beverages, and foods high in sugar. The findings of this study were consistent with global data suggesting that the COVID-19 pandemic had a significant effect on lifestyle including eating habits. Monteiro et al. showed that healthier eating habits were adopted by university students during the lockdown period compared to a typical semester period. Their self-reported healthier eating habits included limited application of meal delivery platforms, less consumption of fast food, pre-cooked meals, foods high in sugar and salt, and sugar-sweetened beverages, more consumption of fruits, vegetables, and legumes, as well as more serious engagement in physical activities (34). Enriquez-Martinez et al. also conducted a study on 6,325 participants aged over 18 years from five countries, and demonstrated that more favorable patterns of eating choice were observed among those who changed their eating habits compared to those who did not change their eating habits (35). However, our study findings were inconsistent with the results from some other studies in this regard. In the study by Robinson et al., a higher number of the participants reported more negative changes in their eating habits (e.g., more frequent snacking) after the lockdown compared to the pre-lockdown period (33). A study by Cheikh Ismail et al. also found that majority of the adults did not eat fruits and vegetables on a daily basis; similar to our study, however, their study showed that the number of consumed meals per day as well as the daily consumption of homemade meals significantly increased during the lockdown compared to the pre-pandemic period (28). A Portuguese study showed that the majority of the participants consumed more foods rich in fat, salt, and/or sugar (e.g., fast food, snacks, sweets, and sugar-sweetened beverages) during the confinement period (7). Another Portuguese study, however, found that 58.2% of the participants increased the consumption of fruits and vegetables but reduced the consumption of pre-cooked meals and sugar-sweetened beverages (36). Similar studies carried out in other countries highlighted the improvement in nutritional habits including increased intake of fruits, vegetables, yogurt, legumes, and eggs during the lockdown (13, 15, 24, 25, 34, 37–41).

Based on our study results, the main causes of the changes in eating habits during the COVID-19 pandemic compared to pre-pandemic period were associated with eating out less frequently, having fear of coronavirus spread by food, preferring home-cooked food, and improving the knowledge about nutrition. These findings may have been attributed to spending more time indoors, working from home, receiving tele-education, and engaging in restricted outdoor physical activities due to the COVID-19 pandemic (41–43). Since people stayed homes for longer time, they may have spent more time to cook homemade meals as well as paid more attention to their diet and, particularly, to the nutritional recommendations proposed for this time period (44). Another factor is concerned with nutrition because most people are aware of the fact that a healthy lifestyle considerably improves the immune system which, in turn, could enhance the likelihood of surviving the virus (45). Sedentary habits mean replacing activity with sleeping, which substantially changes the lifestyle (46), and sleep could be a risk factor for obesity (47). Furthermore, disruption of routine activities due to quarantine could lead to boredom which is connected with higher energy intake (42). Exposure to stressful news about COVID-19 might be associated with overeating and, especially, high-sugar food intake (i.e., “food cravings”) (48).

The physical activities of our participants changed during COVID-19, so that participation in moderate-intensity aerobic exercises for 30 min and leisure-related activities decreased, while participation in household chores, sitting at work for less than 2 h every day, keeping sitting during office hours, watching television for >5 h every day, using mobile phones and social media, as well as playing video games increased. Generally, our university students reported that their participation in physical activities decreased, and their screen and sitting time increased during the pandemic. Our study findings were confirmed by most observational studies reporting that physical activity levels decreased, but screen/sitting time and household activity levels increased (20, 30, 40, 41, 49). For example, Abouzid et al. investigated 5,896 participants aged ≥18 years and demonstrated that nearly 38.4% of the individuals stopped physical activities and exercising during the COVID-19 confinement, and 57.1% of them spent more than 2 h on following social media (40). Chopra et al. also reported that the physical activity levels decreased while the daily screen time increased (20). Our study participants spent more time on walking, working out at home, and engaging in video-based activities in order to increase their physical activity levels during the pandemic. They also reported that the lack of motivation as well as the lack of access to sport facilities and gym were the causes of changes in their physical activities during COVID-19 pandemic. Similarly, Sekulic et al. indicated that the most common physical activities among medical sciences students were walking and exercising at home before and during the COVID-19 pandemic, respectively. Moreover, they found that sitting time increased during the pandemic, but the daily time spent on physical activities during the pandemic was not different from that recorded before the pandemic period (41). Enriquez-Martinez et al. conducted a study on 6,325 individuals aged >18 years from five countries and demonstrated that majority of them performed physical activities at homes (35). However, some studies indicated the increase in self-reported physical exercises during the lockdown (7, 15, 34). The longer time spent at home and on online education may increase the screen time and, consequently, decrease the physical activity level. The negative effects of screen time on weight are usually mediated when screen time displaces the time for physical activity. Therefore, if screen time for education is not replaced with important activities, these negative effects can be avoided during the pandemic (30, 50).

The university students participated in this study stated that oversleeping (>8 h) and poor quality of sleep increased during COVID-19 pandemic. The changes in sleeping patterns during COVID-19 were associated with daytime sleeping, stress, and anxiety. Similar to our study, recent studies showed that a poor sleep quality during COVID-19 confinement period may have been associated with higher stress and anxiety (18, 46, 51, 52). Daily stress and anxiety levels were increased among our participants during COVID-19 pandemic, which were associated with worries about family/friends and fear of COVID infection. Chopra et al. demonstrated that stress and anxiety in nearly one-fourth of the participants were related to quarantine, and that mental health was adversely affected by it (20) Moreover, Enriquez-Martinez et al. revealed that the participants over 18 years of age were affected by anxiety (35). Stress and anxiety induced by the COVID-19 lockdown may cause changes in eating habits (7). Another factor resulting in stress and anxiety among students is fear and concern over their health and loved ones (53). In addition, the lack of social interaction, particularly among active or young individuals, can induce anxiety, stress, and depression. The isolation feelings may stimulate emotional eating for relieving negative feelings (42). Familiarity with these factors facilitates developing effective interventions for mitigating the negative lifestyle behaviors during the COVID-19 pandemic. As for smoking, 2.2% of our participants started smoking, and it did not decrease during COVID-19 pandemic. Berlin et al. have shown that social isolation and mental distress might increase the false need for smoking (40). In the study by Di Renzo et al., however, 3.3% of the smoker respondents decided to quit smoking (15).

It is generally believed that healthy eating and physical activities, particularly during the immune system’s challenging times, are the key factors for improving health and well-being (54). A healthy diet and eating habits together with weekly exercise can help the individuals to fight against the viral diseases such as COVID-19 (54). Worries about the one’s health or the loved ones’ health contribute to greater monitoring of one’s health and personal hygiene as well as to paying more attention to the nutritional value and quality of foods (25). In this study and earlier studies, it was shown that the healthy eating was widely practiced during the pandemic. However, low physical activity levels, stress and anxiety, and tediousness due to social isolation may have negatively affected the lifestyle patterns (55).

### The strengths and limitations

The current research is the first study that evaluated the effects of the COVID-19 pandemic on the lifestyle of college students, however, this study had some limitations. First, the sample size was relatively small and the design was cross-sectional. Second, simultaneous collection of before and after pandemic data. Third, the sample came from various study fields and grades. Fourth, the questionnaire used for collecting data was validated for the population of another country, and the collected data were self-reported.

## Conclusion

In sum, university students stated that their eating habits improved during the COVID-19 pandemic; however, they reported increased weight gain, screen, sitting, and sleeping time, declined physical activities, poor sleep quality, and daily stress or anxiety during the pandemic. Therefore, although the COVID-19 pandemic led to some adverse effects on participants’ lifestyles, the eating habits of university students improved likely due to concerns about their health and efforts to contain COVID-19. Our study’s findings may have had some implications for public health policy. We can use the reported healthy eating habits influencing factors reported by university students during the COVID-19 lockdown to promote better nutrition behaviors outside of the lockdowns. It was recommended that future longer follow-up studies on both genders should be conducted to more examine the relationship between the COVID-19 pandemic and eating habits.

## Data availability statement

The original contributions presented in this study are included in the article, further inquiries can be directed to the corresponding author.

## Ethics statement

The studies involving human participants were reviewed and approved by the Ethics Committee of Tabriz university of medical sciences (ethical cod: IR.TBZMED.REC.1401.597). The patients/participants provided their written informed consent to participate in this study.

## Author contributions

RM-G and MR wrote the main manuscript text. MS contributed to data collection. All authors contributed to the article and approved the submitted version.

## Funding

The research protocol was approved and supported by the Student Research Committee, Tabriz University of Medical Sciences, Tabriz, Iran (grant number: 68993).

## Conflict of interest

The authors declare that the research was conducted in the absence of any commercial or financial relationships that could be construed as a potential conflict of interest.

## Publisher’s note

All claims expressed in this article are solely those of the authors and do not necessarily represent those of their affiliated organizations, or those of the publisher, the editors and the reviewers. Any product that may be evaluated in this article, or claim that may be made by its manufacturer, is not guaranteed or endorsed by the publisher.
